# The influence of adolescents essential and non-essential use of technology and Internet addiction on their physical and mental fatigues

**DOI:** 10.1038/s41598-024-51655-x

**Published:** 2024-01-19

**Authors:** Maryam Aziz, Khansa Chemnad, Sanaa Al-Harahsheh, Azza O. Abdelmoneium, Ahmed Bagdady, Diana Alsayed Hassan, Raian Ali

**Affiliations:** 1 College of Science and Engineering, Hamad Bin Khalifa University, Ar-Rayyan, Qatar; 2 World Innovation Summit for Health, Qatar Foundation, Doha, Qatar; 3 Doha International Family Institute, Qatar Foundation, Doha, Qatar; 4 World Innovation Summit for Education, Qatar Foundation, Doha, Qatar; 5 Department of Public Health, College of Health Sciences, QU Health, Qatar University, Doha, Qatar

**Keywords:** Human behaviour, Psychology and behaviour

## Abstract

This study explores the impact of Internet addiction (IA), age, and essential and non-essential technology usage time on the physical and mental fatigue of adolescents. The research surveyed 477 adolescents from Qatar and employed the Internet Addiction Diagnostic Questionnaire (IADQ) and Chalder's Fatigue Scale for data collection. Multiple linear regression and Mann–Whitney U tests were utilized for analysis. The findings indicate that IA, non-essential usage time, and age are positively associated with overall fatigue among adolescents. IA and non-essential usage time are positively linked to physical fatigue, while IA, non-essential usage time, and age are positive predictors of mental fatigue. However, essential usage time is negatively associated with mental fatigue. These results highlight the importance of distinguishing technology usage based on intent and necessity, as well as differentiating between physical and mental fatigue when examining problematic technology usage. This study is among the few conducted in the Middle Eastern context.

## Introduction

With the widespread availability and essentiality of the Internet, there have been growing public health concerns related to the implications of the excessive use of internet, such as internet addiction (IA), in addition to other mental and physical health related issues^1^. While there is no standard definition of IA, it is generally characterized by excessive and uncontrolled use of the Internet accompanied by different symptoms of withdrawal and dysfunctional coping in addition to detrimental physical and psychological outcomes^2^. The prevalence of IA amongst the general population varies by geographical regions and assessment tools used. A meta-analysis on the prevalence of IA in 31 nations found Asian countries to have higher rates of IA as compared to other regions^3^. Additionally, Middle Eastern countries were at the top with IA rates of 10.9% while Northern and Western European countries were found to have the lowest rates of IA at 2.6%^3^.

Although Internet addiction has not been formally recognized as a behavioral disorder; there is evidence from the field of mental health suggesting that internet addiction may manifest symptoms similar to substance abuse and pathological gambling^4^. A study that examined the psychological effects of ceasing internet use on problematic internet users, found that similar effects were seen in individuals who ceased using sedatives or opiates^5^. Participants experienced withdrawal-like symptoms including an increase in anxiety levels, heart rate and systolic blood pressure^5^. Tonioni et al.^6^ found that individuals with an internet addiction shared similar depression and anxiety levels symptoms with the pathological gambling group in a clinical study. However, individuals with IA were more socially disengaged compared to the pathological gambling group. Further, individuals with IA can also be with other maladaptive behaviors such as impulsivity, aggressiveness^7^, nicotine use disorder, alcohol use disorder, pathological gambling, and suicidality^8^.

Adolescents are primarily affected by IA compared to other age groups. This is due to their developing brains’ sensitivity to signals of excitement thereby making it difficult for them to control their Internet use^9^. Studies on adolescence’ IA found different personal, family, and environmental factors associated with adolescent internet addiction^7^ including gender^10–12^, parent’s IA^12–14^, parenting styles^12,13^, family dynamics^10,11^, school performance^12^ and parents’ and peers’ influence^15^. A cross-sectional survey found that IA mediated the relationship between social anxiety and poor psychosocial wellbeing in adolescents^16^. This signifies that while Internet use may be seen as a relief and escape from the outside world, engaging in it excessively may be a contributing source towards psychological health conditions in adolescents.

While there is no doubt that technology plays a critical role in everyone’s lives in all disciplines, the rapid adoption of technology can also have negative effects, especially on health. Literature has shown there is a relationship between excessive technology use and fatigue. Sleep quality is a main factor in mental and physical fatigue^17^. Sleep patterns are often negatively impacted with users staying up late at night on their screens^18^. A cross-sectional study by Liang et al.^19^ found fatigue to be positively associated with IA in a sample of college students. Another study among students in Turkey, investigated the relationship of IA with fatigue and lifestyle factors. Their results showed that fatigue, irregular sleep, and poor dietary habits were associated with IA in university students^20^. Disrupted sleep patterns and excessive nighttime activities expose individuals to an increased risk of fatigue with adolescents being at a higher risk of impact comparatively^18^. Fatigue is defined as the low level of energy and increased exhaustion that one may experience after completing everyday activities^21^. Individuals experiencing fatigue for longer than six months with additional physical and mental symptoms are often diagnosed with chronic fatigue syndrome^22^. For adolescents and children, a shorter period of three months is required along with one additional symptom^22^. The increased levels of fatigue can impact both physical and cognitive conditions in adolescents.

Fatigue levels in adolescents in the USA were reported as 40%^23^; however, there is limited research on the risk factors of fatigue in adolescents. A survey conducted among adolescents in the USA found a large number of adolescents reported morning fatigue with females and older adolescents at a higher risk of fatigue^24^, With IA impacting users’ lifestyle aspects such as sleep and dietary habits, fatigue levels may also be impacted. A cross-sectional study by Bener^25^ examined the lifestyle factors affecting adolescents IA in Qatar in 2011–2012. The study found that adolescents’ IA, with a prevalence of 19.8%, was positively associated with fatigue. With Qatar having one of the highest internet penetration rates in the world at 99%^26^ coupled with the massive increase in handheld devices, internet users in Qatar have increased from 69% in 2012 to almost 100% in 2022^27^. Additionally, with the increasing number of new apps continuously being introduced in the marketplace, the IA prevalence rates may have also increased. Qatar experiences hot and humid in almost half of the year. As a result, adolescents and families can be more inclined to indoor activities such video gaming and other forms of technology use.

Different stages of adolescence may also impact fatigue as adolescents go through rapid developments in both biological functioning and social environments. The stage of early adolescence is identified as between 10 and 13 years whereas middle adolescents is identified as between 14 and 17 years^28^. A study by Cerniglia et al.^29^ reported that the severity of IA, depression and anxiety often increases from early to late adolescence with major differences seen from early to middle stages of adolescence. Further, changes in school settings and academic challenges may also lead to differences in fatigue amongst early and middle adolescents^30^. Middle adolescents face higher expectations from their parents as compared to early adolescents. With the shift from early to late adolescence, adolescents become more susceptible to symptoms of depression, insomnia, and anxiety. A survey on early, middle, and late adolescents found depression and insomnia mediating the relationship between IA and academic engagement with the relationship stronger in middle and late adolescents than early adolescents^31^.

According to Henrich et al.^32^, the majority of study samples in the area of psychology consist of individuals who are western, educated, industrialized, wealthy, and democratic (WEIRD). Despite this, there have been few attempts in practice to enhance diversity, as highlighted by Medin et al.^33^. This underrepresentation of the majority of the world's population is especially evident in fields like developmental psychology, where Euro-American samples dominate most studies^34^. This study on adolescent internet addiction offers a significant contribution to the field of psychology by presenting novel findings from a sample that is not restricted to WEIRD individuals and is conducted in Qatar. The research examines the impact of IA and technology usage time on fatigue, with a unique focus on differentiating between essential and non-essential usage and considering both types of fatigue, mental and physical. These distinct features of our study offer valuable insights into the complexities of problematic technology usage among adolescents.

Adolescents’ use of digital technology may be of two types: essential and non-essential. Adolescents may use the internet for essential daily tasks such as for educational purposes or work. For the non-essential use, internet is used for purposes of passing time and pleasure, e.g., social media, games, and other entertainment sites^35^. Researchers have examined the relationship between excessive internet use, internet addiction and fatigue, however; studies do not tend to distinguish between essential versus non-essential internet use. We argue that utilizing the Internet to complete necessary tasks that are important for everyday life, work, and school saves time, increases efficiency, and in turn reduces fatigue. On the other hand, the overuse of the Internet, especially by engaging in non-essential activities may lead to reduced productivity, poor sleeping habits and ultimately fatigue. Therefore, one of our study aims is to gain a better understanding if the two different types of internet use are linked with fatigue. In this paper, we aim to answer the following research questions:

- (RQ1) Do the times spent on digital technology for essential and non-essential purposes, IA level, and age predict mental and physical fatigues among adolescents?
- (RQ2) Do the individual symptoms of IA predict physical and mental fatigue among adolescents?

## Method

### Participants

An online survey was sent out to 16 public and private schools in Qatar between March 2022 and May 2022. The study was approved by the Institutional Review Board (IRB) of the first author’s institution and school permissions were obtained to distribute the survey. The study was conducted according to the principles set forth in the Declaration of Helsinki. Participation in the study was voluntary. Informed consent was obtained from all the study participants. The parents and adolescents were informed beforehand of the study and their explicit consent and assent were requested before starting the survey. 477 completed the survey to the end. After cleaning the data, the final sample had 331 participants.

We used Green's formula^36^ to determine the appropriate sample size for our study. This formula suggests that a minimum sample size of 50 + 8 times the number of independent variables *p* is needed for a linear regression analysis. This suggests that a minimum sample size of 82 participants is appropriate for our study. Furthermore, to apply correlation stability^37^, we aimed for a sample size greater than that of 250 participants.

### Measures

The survey collected information regarding participants’ demographics, digital technology usage and IA. Data regarding participants’ family environment and health status were also gathered. Additionally, the survey was administered in both English and Arabic (see Supplementary Materials for both versions of the survey). The Arabic survey was generated using the back-translation method to ensure the quality of translation and the preservation of the original meaning is maintained.

#### Internet addiction diagnostic questionnaire (IADQ)

The Internet Addiction Diagnostic Questionnaire (IADQ) was developed to measure an individual’s level of internet addiction. Due to the lack of available diagnostic criteria for IA, the questionnaire was developed by Young^38^ based on the pathological gambling criteria since it was found to be the closest to IA in DSM-IV. The IADQ is comprised of eight closed-ended questions (yes/no) where the total score of the questionnaire is calculated by the number of questions the participant responded "yes" to. Each question represents a different symptom of IA. These symptoms are preoccupation with internet, tolerance, unsuccessful efforts to control Internet use repeatedly, withdrawal, staying online longer than intended, risk/loss of relationships and opportunities because of the Internet use, lies to conceal the extent of involvement and dysfunctional coping. Participants are asked to answer the questionnaire based on their non-essential internet use, that is, non-business or non-academic use. The IADQ total score ranges from 0 to 8, where Young^38^ suggests that a score of 5 or more classifies the participant as a dependent internet user whereas a score below 5 classifies the participant as a non-dependent Internet user. Previous literature on IADQ has found Cronbach’s alpha to be within the range of 0.60 and 0.72^39^. Cronbach’s alpha for IADQ for this study was 0.66 indicating an acceptable level of reliability^40^.

#### Chalder fatigue scale (CFQ-14)

The Chalder Fatigue Scale is comprised of 14 items that determine the physical and mental fatigue levels of an individual^41^. The scale measures physical fatigue symptoms using the first eight questions (1–8) while the mental fatigue symptoms are measured using the remaining six questions (9–14). The questionnaire uses a 4–point Likert scale where 1 = better than usual, 2 = no more than usual, 3 = worse than usual and 4 = much worse than usual. For physical symptoms, the scores from question 1 to 8 are summed together and for mental symptoms, the remaining six questions are summed. The participant can accumulate a maximum possible score of 56 on the total fatigue scale with a higher score indicating higher fatigue levels. The physical scale total score can range from 8 to 32 and the mental scale score can range from 6 to 24. Cronbach’s alpha for the CFQ-14 scale was measured at 0.88–0.90 for total fatigue, 0.845 for physical fatigue and 0.821 for mental fatigue. The Cronbach’s alpha for the total fatigue, physical fatigue and mental fatigue scales in this study were 0.90, 0.85 and 0.81 respectively indicating a good level of reliability for all three^40^.

While the CFQ-14 has been used for chronic fatigue syndrome amongst other medical conditions, it can be administered alongside IADQ to provide a thorough assessment of individuals experiencing fatigue in relation to their IA as seen in previous literature^19,42^. Bener et al.^20^ studied the association between fatigue levels and IA levels in a group of Turkish university students using the CFQ-14 to measure fatigue levels and the Internet Addiction Test (IAT), a variation of IADQ to measure the IA levels. Another study on female nurses used the Chen Internet Addiction Scale with CFQ-14 to study association between fatigue and IA in hospital nurses^43^. It is also worth noting that the CFQ-14 encompasses both mental and physical fatigue and this broad measurement can be considered to be a methodological strength, as it allows us to establish a link between IA and life fatigue, rather than restricting our focus to narrow concepts such as technostress^44^.

#### Digital technology use

Participants were requested to provide their time spent on essential and non-essential use of digital technology on both weekdays and weekends. They were asked open-ended questions on how many hours they spent on digital technology on the weekdays (Sunday–Thursday) and on the weekends (Friday and Saturday). The values for essential and non-essential use provided were cleaned for consistency and missing or incomprehensible values were removed. For essential use of digital technology on weekdays, the average of the value was taken for users who provided a range. For example, users who mentioned that they spent 7 to 8 h on digital technology during weekdays, we took the essential use as 7.5 h. A few participants also mentioned their weekly usage instead of daily and hence, their answer was averaged over 5 days for the weekdays. Similar cleaning steps were taken for non-essential use on weekdays, essential use on weekends and non-essential use on weekends. For weekends, however, the answers were averaged over 2 days instead of 5 days where necessary. The participants’ essential use and non-essential use were then calculated by summing their weekdays and weekends use together.

### Data analysis

The statistical analysis for this study was performed using JASP 0.16.3^45^. Linear regression was used to determine the relationship between total fatigue and the different predictors. Furthermore, for determining the relationship of physical and mental fatigue with the different predictors, linear regression was used. Assumption checks for each analysis showed that the criteria of linearity, normality, and autocorrelation for conducting linear regression were met^46^. The VIF for the independent variables in each model was within the range of 1–2.5 thereby suggesting the lack of multicollinearity between the independent variables^47^. The individual symptoms of IA were also tested against physical and mental fatigue to determine their relationship. Mann Whitney U-test was conducted due to the lack of normality in each group of the sample. Cohen’s d was used to determine the effect size of the relationships. Statistical significance was accepted at *p* < 0.05.

### Ethical statement

The study was approved by the Institutional Review Board (IRB) of Hamad Bin Khalifa University, Qatar.

## Results

Responses of 586 students were recorded of which 477 students completed the survey to the end. Missing values were found in responses to essential and non-essential use of digital technology. Some participants also gave implausible values such as daily non-essential use that is more than 24 h or unquantifiable responses such as “little” or “too much”. Missing values along with these implausible and unquantifiable values were removed from the sample. The sample also contained records of only two late adolescents and due to the small sample size in the age group, the two records were removed to reduce noise in the sample. The final sample had 331 participants with the mean age of 13.2 years (SD = 1.23). The sample consisted of 260 (78.5%) females and 71 (21.5%) males. Two of the schools with the most respondents to the survey were predominantly female schools. In addition, age as a continuous variable was also measured using open-ended questions. The sample also included 211 early adolescents (10–13 years old) and 120 middle adolescents (14–17 years old). Of the middle adolescents, only two participants were aged 17 years.

### Descriptive statistics

Table 1 summarizes the descriptive statistics of the sample for this study. Approximately 79% of the participants were females whereas 21% were males. The participants were all residents of Qatar, with 91% from Middle Eastern countries and 9% from Western countries.

**Table 1:** Descriptive Statistics of sample variables.

Parameters	Mean	Std. dev
Age	13.20	1.23
Essential daily use (hours)	2.43	1.81
Non-essential daily use (hours)	4.47	2.62
Total fatigue	28.59	9.30
Physical fatigue	16.09	5.51
Mental fatigue	12.50	4.39
IADQ	3.33	2.05
Internet Addiction prevalence	n	%
Addicted	102	30.82
Non-addicted	229	69.18
Individual symptoms of IA prevalence	No	Yes
	n (%)	n (%)
Preoccupation	174 (52.57)	157 (47.43)
Tolerance	236 (71.30)	95 (28.70)
Unsuccessful efforts to control Internet use repeatedly	181 (54.68)	150 (45.32)
Withdrawal	202 (61.03)	129 (38.97)
Staying online longer than intended	127 (38.37)	204 (61.63)
Risk/lose relationships/opportunities because of the Internet	249 (75.23)	82 (24.77)
Lies to conceal extent of involvement	256 (77.34)	75 (22.66)
Dysfunctional coping	120 (36.25)	211 (63.75)

### RQ1: relationship of technology use and IA with fatigue

Multiple linear regression was used to study the relationship between the different predictors for adolescent Internet use and total fatigue and its two subscales: physical and mental fatigue. The first regression analysis included total fatigue as the outcome. Age, total IA, essential, and non-essential uses were the predictors in the model. All the assumptions of multiple linear regression were checked. To test for multicollinearity, Pearson correlation was used which showed the sample did not contain multicollinearity among the predictors as none of the correlations were above 0.8^48^. Table 2 shows the correlation between the variables in the model. Furthermore, Figure S1 in Supplementary Materials shows the scatter plots for the association between the different predictors for adolescent Internet use and total fatigue and its two subscales: physical and mental fatigue.

**Table 2:** Correlation between total fatigue, physical fatigue, mental fatigue, total IA, essential use, non-essential use, and age.

Variable	Pearson’s r						
	Total fatigue	Physical fatigue	Mental fatigue	Total IA	Essential use	Non-essential use	Age
Total fatigue	–						
Physical fatigue	0.95***	–					
Mental fatigue	0.92***	0.76***	–				
Total IA	0.50***	0.46***	0.47***	–			
Essential use	0.037	0.077	− 0.018	0.12*	–		
Non-essential use	0.38***	0.32***	0.41***	0.43***	0.062	–	
Age	0.16**	0.15**	0.15**	0.12*	0.26***	0.055	–

*p < 0.05, **p < 0.01, ***p < 0.001.

The sample also met the assumptions of linearity, normality, and homoscedasticity. The linearity of the sample was checked using residuals vs. predicted plots. Q-Q plots were used to check the normality of the data and residuals vs. dependent plots were used to check for homoscedasticity in the data. The regression model for total fatigue was found to be significant (*F (4, 326)* = *33.73,* p < 0.001, R^2^ = 0.29, adjusted R^2^ = 0.28). Total IA *(β* = *0.40, p* < *0.001)*, non-essential use *(β* = *0.21, p* < *0.001)* and age *(β* = *0.11, p* = *0.022)* were the significant predictors in the model as shown in Table 3.

**Table 3:** Multiple linear regression analysis for total fatigue.

	R^2^	Adjusted R^2^	F (df)
	0.29	0.28	33.73 (4, 326)
Predictors	*Standardized β*	*T*	*p*
Constant		1.86	0.063
Total IA	**0.40**	**7.71**	** < 0.001**
Essential use	− 0.052	− 1.08	0.28
Non-essential use	**0.21**	**4.03**	** < 0.001**
Age	**0.11**	**2.30**	**0.022**

Significant values are in bold.

The second regression analysis involved the predictors of age, total IA, essential use, and non-essential use with the outcome of physical fatigue. All the assumptions of linearity, normality, multicollinearity, and homoscedasticity were met. Residuals vs. predicted plots, Q-Q plots, Pearson’s r and residuals vs. dependent plots were used to check for the respective assumptions. The regression model for physical fatigue was found to be significant (*F (4, 326)* = *26.03,* p < 0.001, R^2^ = 0.24, adjusted R^2^ = 0.23). Total IA *(β* = *0.39, p* < *0.001)*, and non-essential use *(β* = *0.15, p* = *0.005)* were the significant predictors in the model as shown in Table 4.

**Table 4:** Multiple linear regression analysis for physical fatigue.

	R^2^	Adjusted R^2^	F (df)
	0.24	0.23	26.03 (4, 326)
Predictors	*Standardized β*	*T*	*p*
Constant		1.99	0.048
Total IA	**0.39**	**7.26**	** < 0.001**
Essential use	− 0.003	− 0.059	0.95
Non-essential use	**0.15**	**2.80**	**0.005**
Age	**0.092**	**1.84**	**0.067**

Significant values are in bold.

The third regression analysis looked into predicting mental fatigue with age, total IA, essential use and non-essential use as the predictors. All the assumptions of linearity, normality and homoscedasticity were met. Residuals vs. predicted plots were used to check for linearity whereas Q-Q plots were used to check the normality of the data. Residuals vs. dependent plots were used to check for homoscedasticity in the data. The sample did not contain multicollinearity as the correlation between variables was below 0.8 as can be seen in Table 2. The regression model for mental fatigue was significant (*F (4, 326)* = *33.05,* p < 0.001, R^2^ = 0.29, adjusted R^2^ = 0.28). Total IA *(β* = *0.36, p* < *0.001)*, essential use *(β* = *-0.11, p* = *0.028)*, non-essential use *(β* = *0.25, p* < *0.001)* and age *(β* = *0.12, p* = *0.014)* were the significant predictors in the model as shown in Table 5.

**Table 5:** Multiple linear regression analysis for mental fatigue.

	R^2^	Adjusted R^2^	F (df)
	0.29	0.28	33.05 (4, 326)
Predictors	*Standardized β*	*T*	*p*
Constant		1.36	0.174
Total IA	**0.36**	**6.87**	** < 0.001**
Essential use	** − 0.11**	** − 2.20**	**0.028**
Non-essential use	**0.25**	**4.88**	** < 0.001**
Age	**0.12**	**2.46**	**0.014**

Significant values are in bold.

### RQ2: Relationship of individual symptoms of IA and physical and mental fatigue

The Mann Whitney U-test was used to study the analysis of RQ2. Table 6 shows a summary of the Mann Whitney U tests conducted for each of the symptoms with the different fatigue measures.

**Table 6:** Mann–Whitney U test results for fatigue levels in adolescents with IA symptoms.

Symptom	Yes		No			Fatigue measure	U	p	|r|
	n	Mdn	n	Mdn					
Preoccupation	157	29	174	26		Total fatigue	10,239.00	< 0.001	− 0.25
		17		14		Physical fatigue	10,126.50	< 0.001	− 0.26
		13		12		Mental fatigue	10,761.00	< 0.001	− 0.21
Tolerance	95	30	236	27		Total fatigue	8314.50	< 0.001	− 0.26
		17		15		Physical fatigue	8679.50	< 0.001	− 0.23
		13		12		Mental fatigue	8278.00	< 0.001	− 0.26
Unsuccessful efforts	150	29	181	26		Total fatigue	10,035.50	< 0.001	− 0.26
		17		15		Physical fatigue	10,087.50	< 0.001	− 0.26
		13		11		Mental fatigue	1033.50	< 0.001	− 0.24
Withdrawal	129	30	202	26		Total fatigue	9240.50	< 0.001	− 0.29
		17		15		Physical fatigue	9778.00	< 0.001	− 0.25
		14		11		Mental fatigue	9037.50	< 0.001	− 0.31
Staying online longer	204	29	127	24		Total fatigue	7863.00	< 0.001	− 0.39
		17		13		Physical fatigue	8432.00	< 0.001	− 0.35
		13		10		Mental fatigue	7848.00	< 0.001	− 0.39
Losing relationships	82	32.5	249	27		Total fatigue	7269.50	< 0.001	− 0.29
		18		15		Physical fatigue	7158.00	< 0.001	− 0.30
		13		12		Mental fatigue	7807.00	< 0.001	− 0.23
Lying to conceal	75	33	256	26		Total fatigue	5687.00	< 0.001	− 0.41
		19		15		Physical fatigue	5770.50	< 0.001	− 0.40
		14		12		Mental fatigue	6153.00	< 0.001	− 0.36
Dysfunctional coping	211	30	120	23		Total fatigue	6454.00	< 0.001	− 0.49
		17		13		Physical fatigue	7384.00	< 0.001	− 0.42
		13		10		Mental fatigue	6202.50	< 0.001	− 0.51

For the first symptom of preoccupation, total fatigue was found to be significant with adolescents who exhibited preoccupation symptoms (Mdn = 29, n = 157) scoring higher on the total fatigue scale as compared to adolescents who did not (Mdn = 26, n = 174), *U* = 10,239.00, *p* < 0.001, |*r*|= − 0.25. Physical fatigue was also found to be significant, *U* = 10,126.50, *p* < 0.001, |*r*|= − 0.26. Adolescents who exhibited the symptom of preoccupation (Mdn = 17, n = 157) scored higher on the physical fatigue scale as compared to adolescents who did not (Mdn = 14, n = 174). Preoccupation was also a significant predictor of mental fatigue, *U* = 10,761.00, *p* < 0.001, |*r*|= − 0.21. Adolescents with preoccupation symptoms (Mdn = 13, n = 157) scored higher on the mental fatigue scale as compared to adolescents without preoccupation symptoms (Mdn = 12, n = 174).

The second symptom of tolerance was found to be significant with total fatigue, *U* = 8314.50, *p* < 0.001, |*r*|= − 0.26. Adolescents who exhibited the tolerance symptom (Mdn = 30, n = 95) scored higher on the total fatigue scale as compared to those who did not (Mdn = 27, n = 236). Physical fatigue was also significant, *U* = 8679.50, *p* = 0.001, |*r*|= − 0.23 and adolescents who reported the tolerance symptom (Mdn = 17, n = 95) scored higher on the physical fatigue scale as compared to those who did not (Mdn = 15, n = 236). The tolerance symptom was also significant with mental fatigue with adolescents expressing the tolerance symptom (Mdn = 13, n = 95) scoring high on the mental fatigue scale as compared to those who did not (Mdn = 12, n = 236), *U* = 8278.00, *p* < 0.001, |*r*|= − 0.26.

Total fatigue was significant with the third symptom of unsuccessful efforts to control Internet use repeatedly, *U* = 10,035.50, *p* < 0.001, |*r*|= − 0.26. Adolescents who exhibited the third symptom (Mdn = 29, n = 150) scored higher on the total fatigue scale as compared to those who did not (Mdn = 26, n = 181). Additionally, physical fatigue was also significant with adolescents who exhibited the symptom (Mdn = 17, n = 150) scoring higher on the physical fatigue scale than adolescents who did not (Mdn = 15, n = 181), *U* = 10,087.50, *p* < 0.001, |*r*|= − 0.26. Mental fatigue was also found to be significant, *U* = 10,333.50, *p* < 0.001, |*r*|= − 0.24. Adolescents who responded in affirmative to the symptom (Mdn = 13, n = 150) scored higher on the mental fatigue scale as compared to those who did not (Mdn = 11, n = 181).

For the fourth symptom of withdrawal, total fatigue was found to be significant with adolescents who expressed withdrawal symptoms (Mdn = 30, n = 129) scoring higher on the total fatigue scale as compared to adolescents who did not (Mdn = 26, n = 202), *U* = 9240.50, *p* < 0.001, |*r*|= − 0.29. Physical fatigue was also found to be significant for the withdrawal symptom, *U* = 9778.00, *p* < 0.001, |*r*|= − 0.25. Adolescents who replied in affirmative (Mdn = 17, n = 129) scored higher on the physical fatigue scale as compared to those who did not (Mdn = 15, n = 202). Withdrawal symptom was significant with mental fatigue with adolescents who were suffering from the symptom (Mdn = 14, n = 129) scoring higher on the mental fatigue scale as compared to those who were not (Mdn = 11, n = 202), *U* = 9037.50, *p* < 0.001, |*r*|= − 0.31.

The fifth symptom of staying online longer than intended was significant with total fatigue, *U* = 7863.00, *p* < 0.001, |*r*|= − 0.39. Adolescents who responded in affirmative (Mdn = 29, n = 204) scored higher on the total fatigue scale than those who did not (Mdn = 24, n = 127). Furthermore, the fifth symptom was also significant for physical fatigue with those who exhibited the symptom (Mdn = 17, n = 204) scoring higher on the physical fatigue scale as compared to those who did not (Mdn = 13, n = 127), *U* = 8432.00, *p* < 0.001, |*r*|= − 0.35. Mental fatigue was also found to be significant with the fifth symptom, *U* = 7848.00, *p* < 0.001, |*r*|= − 0.39. Adolescents who expressed staying online longer than intended (Mdn = 13, n = 204) scored higher on the mental fatigue scale as compared to those who did not (Mdn = 10, n = 127).

The sixth symptom of risk of losing relationships and opportunities because of the Internet was significant with total fatigue and adolescents who exhibited the symptom (Mdn = 32.5, n = 82) scoring higher on the total fatigue scale as compared to those who did not (Mdn = 27, n = 249), *U* = 7269.50, *p* < 0.001, |*r*|= − 0.29. Additionally, physical fatigue was also significant with the sixth symptom and adolescents who replied in affirmative (Mdn = 18, n = 82) scored higher on the physical fatigue scale than those who did not (Mdn = 15, n = 249), *U* = 7158.00, *p* < 0.001, |*r*|= − 0.30. Mental fatigue was also significant with the sixth symptom, *U* = 7807.00, *p* = 0.001, |*r*|= − 0.23. Adolescents who expressed being at risk of losing relationships (Mdn = 13, n = 82) scored higher on the mental fatigue scale as compared to those who did not (Mdn = 12, n = 249).

For the seventh symptom, total fatigue was found to be significant with adolescent lying to conceal the extent of their involvement in the Internet (Mdn = 33, n = 75) scoring higher on the total fatigue scale as compared to those who did not (Mdn = 26, n = 256), *U* = 5687.00, *p* < 0.001, |*r*|= − 0.41. Physical fatigue was also found to be significant with the seventh symptom and those who responded in affirmative (Mdn = 19, n = 75) to the symptom were found to score higher on the physical fatigue scale as compared to those who did not (Mdn = 15, n = 256), *U* = 5770.50, *p* < 0.001, |*r*|= − 0.40. Additionally, mental fatigue was also found to be significant for the seventh symptom with adolescents who exhibited the symptom (Mdn = 14, n = 75) scoring higher on the mental fatigue scale as compared to those who did not (Mdn = 12, n = 256), *U* = 6153.00, *p* < 0.001, |*r*|= − 0.36.

The final symptom of dysfunctional coping was significant with total fatigue and those who responded in affirmative (Mdn = 30, n = 211) to the eighth symptom were found to score higher on the total fatigue scale as compared to those who did not (Mdn = 23, n = 120), *U* = 6454.00, *p* < 0.001, |*r*|= − 0.49. Furthermore, physical fatigue was also found to be significant, *U* = 7384.00, *p* < 0.001, |*r*|= − 0.42. Adolescents who exhibited the symptom (Mdn = 17, n = 211) scored higher on the physical fatigue scale as compared to those who did not (Mdn = 13, n = 120). Mental fatigue was also found to be significant with the eighth symptom and adolescents expressing dysfunctional coping (Mdn = 13, n = 211) scored higher on the mental fatigue scale than those who did not (Mdn = 10, n = 120), *U* = 6202.50, *p* < 0.001, |*r*|= − 0.51.

In our analysis, we conducted eight Mann–Whitney U tests to examine the relationship between each of the eight symptoms and various measures of fatigue. To account for multiple comparisons and reduce the possibility of Type I errors, we applied the Bonferroni correction. The corrected alpha value was set at 0.00625, which is derived by dividing the standard alpha level (p < 0.05) by the total number of tests conducted (eight). As a result, each test remained significant at the Bonferroni-corrected significance level. The majority of the effect size in our results are at moderate level and fall within the range commonly associated with meaningful practical significance.

## Discussion

This study investigates the relationship between fatigue and different predictors related to Internet use. With adolescents’ IA levels increasing from 19.8% in 2011 to 30.8% in 2022^25^, research on its impact on adolescents’ fatigue levels is essential to understand the extent of the problem. Furthermore, this study also highlights the influence of the essential and non-essential use of the Internet on fatigue levels.

The findings from this study show that physical and mental fatigue are predicted by IA, and non-essential Internet use. Our findings align with the study by Bener^25^ showing IA is positively related with fatigue in adolescents. Furthermore, a study by Dol^49^ found fatigue and pain related to fatigue were associated with internet use duration amongst a sample of university students. The longer duration students spent online, the higher the self-reported fatigue especially on the eyes. Cao et al.^50^ conducted a study on IA in Chinese adolescents and its negative impacts on the adolescents’ physical and psychological health. Their results showed that adolescents who scored high on the IA scale were more likely to report physical and psychological health issues including lack of physical energy, emotional symptoms, behavioral symptoms, and physiological dysfunction. Furthermore, these adolescents reported their Internet use as mostly for entertainment and socializing purposes. The excessive Internet use in adolescents may also be due to the phenomenon of fear of missing out (FOMO). According to Gupta & Sharma^51^, FOMO is a complex phenomenon underpinned by addiction, thus possibly triggering excessive internet use in adolescents. A study by Bloemen & de Coninck^52^ found social media use to be directly related to FOMO in adolescents. FOMO may lead adolescents to spend more time on the Internet trying to stay informed on others’ lives making it difficult for them to get away from their digital devices^53^ and thereby, impacting their fatigue levels. Additionally, sleep quality is impacted by the time spent on Internet where reducing the time spent could help improve sleep quality^54^. This may also impact fatigue levels since disrupted sleep patterns cause increased fatigue levels in adolescents^18^.

Our findings also showed age to be positively related to mental fatigue levels in adolescents. Since our sample contained early and middle adolescents only, this may imply that middle adolescents are at a higher risk of fatigue than early adolescents. This may be due to the increased use of digital technology and reduced parental monitoring as adolescents become older. A study by Richards et al.^55^ reported parental monitoring as a mediator in between problem behaviors and age in adolescents where older adolescents reported less parental monitoring compared to younger adolescents. A review on the relationship of sleep and electronic media use such as the Internet, online gaming, and music, found electronic media use as related to delayed sleep and shorter bedtime thereby impacting the quality of sleep of adolescents^56^. A longitudinal study on adolescents and risk behavior found parental monitoring to be inversely related to risk behaviors. Additionally, perceived parental monitoring had long-term effects on adolescents who were less likely to be involved in risk behaviors as they aged as compared to adolescents who had lesser perceived parental monitoring^57^. Similar to our study, their sample also contained a majority of female participants. A study examining fatigue severity in adolescents found age to impact fatigue levels in females whereas it had no effect in males^58^. The study further stated that females tend to go through developmental changes from middle adolescence hence, experience mental changes and are at a higher risk of fatigue.

One of the interesting results of our study is the negative relationship of mental fatigue with the essential use of digital technology. While studies have looked into the use of digital technology for non-essential reasons such as online gaming, Internet use, music, and social media use, limited research has been done on the essential use of digital technology and its contribution towards adolescents’ health conditions. The findings of this study show the need to also investigate essential use when discussing digital technology use of adolescents since with the social isolation factor and education and learning shifting more towards online resources, essential digital technology use is becoming more significant in adolescents’ everyday lives. With adolescents, essential use is predominantly school-related work assigned by teachers which would occur mostly during school hours and not disrupting adolescents’ sleep patterns. This may explain why essential digital technology use has a negative relation with mental fatigue. A cross-sectional study conducted on the academic performance and purpose for internet use in Korean adolescents found individuals who used the Internet for study purposes were more likely to be high academic achievers^59^. The study further stated that individuals who used the Internet mostly for general purposes of gaming, social networking and blogging may have an imbalanced circadian rhythm due to the interference on the excessive use of Internet with their sleep and schoolwork.

In relation to the individual symptoms of IA, it is interesting to note that all symptoms of IA were significant predictors of fatigue, both mental and physical. One explanation for this could be the coping mechanisms adopted by adolescents. Coping mechanisms are strategies that people use to handle internal and external stressful situations^60^. These mechanisms could include adaptive and maladaptive forms. Maladaptive coping refers to disengagement, avoidance, suppressing emotions, and denial, which could all lead to poor health outcomes^61^. There is evidence suggesting that adolescents with excessive internet use issues tend to use maladaptive forms of coping. In our study, approximately 64% of surveyed adolescents reported engaging in dysfunctional coping mechanisms, the most prevalent IA symptoms among adolescents in our sample. A study that examined problematic internet gaming found that adolescents exhibiting symptoms of Internet Gaming Disorder were positively related to maladaptive coping specifically denial and behavioral disengagement^62^. When adolescents engage in maladaptive coping behaviors, which could include substance abuse or addictive behaviors, it creates a vicious cycle of physical symptoms that contribute in turn to added stress and fatigue. Additionally, adolescents with dysfunctional coping may turn to the Internet as an escaping mechanism from their stressful environment thus contributing to that continuous cycle. Similar studies that examined the use of social media platforms found that the relationship of excessive use of social media can be reciprocal with other mood related disorders such as depressive symptoms, loneliness, and a lower life satisfaction^63–65^. The evidence supporting a similar reciprocal relationship between fatigue and IA is scarce. However, given that fatigue is a common side effect and could be a residual symptom of depression^66^, it warrants that future research studies examine the interactions between IA, fatigue, and mood related disorders. This also includes the population of this study as literature showed a high prevalence of depressive symptoms among adolescents in Qatar^67^.

Two common and related symptoms of IA are staying online longer than intended and preoccupation; the second and third most prevalent symptoms, respectively, in our sample of adolescents. Shapira et al.^68^ proposed that internet addiction should be classified under impulse control disorders under DSM-IV-TR, which further broadened the diagnostic criteria for harmful internet use. Shapira et al.^68^ further explained that maladaptive preoccupation with the internet can be indicated by at least one of the following: (1) Preoccupations with use of the internet that are characterized by irresistible experiences and (2) Excessive use of the internet for periods of time longer than intended. It is documented that preoccupation with internet use may cause clinically significant distress or impairments in social relationships, losing various opportunities, or other important areas of life of individuals affected by this addiction^69,70^. Furthermore, adolescents experiencing such consequences can be exacerbated by sleep deprivation due to the long hours they spend on the internet; all of which are valid contributors to fatigue.

Unsuccessful efforts to control the behavior repeatedly, in addition to lying to conceal the extent of involvement in the addictive behaviors are common traits among individuals with behavioral addictions and substance addictions^71,72^. Individuals who are pathological gamblers for example, tend to lie to hide the extent of their gambling addiction^73^. Given the extensive cognitive efforts, the emotional toll of attempting to conceal the truth about their addiction, and the added stress, adolescents can become fatigued. Consequently, adolescents who are fatigued may have a harder time concentrating and fulfilling their responsibilities causing them to inadvertently lie.

As seen in our study, symptoms of tolerance and withdrawal predicted fatigue. Tolerance, defined as an increase in the amount of time or intensity needed on the internet to achieve pleasure and satisfaction^74^. As stated with the previous IA symptoms, tolerance resulting in prolonged time spent online and away from social interactions may result in negative consequences such as reduced physical activity and consequently fatigue. When individuals with IA are without the internet, they may feel dysphoric, anxious, irritable, and bored. All of which are symptoms of withdrawal^74^. According to evidence, individuals with drug addictions may share neural mechanisms with videogame playing. The dopamine reward mechanism is sub sensitive in Internet addiction, just as it is in drug and alcohol abuse^75^. This may indicate that withdrawal symptoms related to IA mimic the withdrawal symptoms seen in those with drug addiction, one of which is fatigue.

While it is important to examine individual traits and factors related to IA, it is important to consider the cultural and local context in our study as there are certain unique contextual environmental determinants that may favorably contribute to both IA and fatigue. Qatar has one of the highest internet penetration rates in the world at 99% of the total population^26^. The culture of convenience in addition to the hot weather during most of the year, dictates that people spend most of their time indoors.

The main limitation of our study is that it was a cross-sectional study design, which prevented us from examining causal relationships between the variables studied. Additionally, our data was self-reported by adolescents. Factors like recall bias or social desirability may have affected the quality of the data. For example, students may not have disclosed the true or correct number of hours they spent online. While evidence suggests that gender-based differences may exist in adolescents’ internet addiction, our sample included 80% females: thus, hindering us from examining these differences. Additionally, due to administering the survey during exam time, it impacted the willingness of the schools to distribute the survey to the students resulting in a smaller sample size than we originally desired. We also note the reciprocal relationship between mental and physical fatigue, where one can influence the other. To gain a comprehensive understanding, it is worth considering the inclusion of both types of fatigue in the regression model predicting each other. This approach could provide valuable insights into the impact of additional variables (e.g., age, usage time, and IA) on the outcome variable. However, it is important to address the collinearity issues arising from the correlation between the two types of fatigue. High correlation levels between a predictor (one type of fatigue) and the outcome (the other type of fatigue) can lead to overfitting, an inflation of R-Square, uncertainty regarding the impact of other predictors, and potential redundancy. To distill the specific impact of each type of fatigue, a different study design is recommended. This might involve a sample of participants representing various levels of both fatigue types and different correlation levels.

Despite these limitations, our study shed light on IA and fatigue; two very important issues that have been affecting adolescents. Our study found that almost a third of our sample exhibited symptoms of IA, which is alarming. Our study also found that Internet use, specifically non-essential use, and IA are linked to fatigue. We found that every symptom of IA is a predictor of fatigue; both mental and physical. While we examined IA as a behavioral addiction and as a predictor of fatigue, we acknowledge that in order to gain a holistic and comprehensive understanding of these issues affecting adolescents, future studies must focus on adopting a multi-level framework to allow investigating beyond personal factors including environmental and systems-related factors contributing to or mediating internet addiction and fatigue among adolescents. This will pave the path for a comprehensive and a holistic planning approach for interventions and preventive efforts.

### Supplementary Information

Supplementary Information.

## Data Availability

Data supporting the findings of this study are available from the corresponding author on request.
