# Supplementary material for: The influence of adolescents essential and non-essential use of technology and Internet addiction on their physical and mental fatigues
Source: Sci Rep. 2024 Jan 19;14:1745. doi: 10.1038/s41598-024-51655-x (PMC10799030; doi:10.1038/s41598-024-51655-x)
Supplement: Supplementary file 1 — Supplementary Information. [file 41598_2024_51655_MOESM1_ESM.docx]

**Supplementary Materials**

1. **Survey – English version**

**Excessive Use of Digital Technologies – Research Project**

**Children Survey**

This study is on the “What impact does excessive use of digital technologies have on family cohesion and relationships within the family, health of children and adults, and student learning, and what should parents and policymakers do to address this issue in Qatar.”

You are participating in this study on a voluntary basis. You can withdraw from the study at any point without having to give reasons and with no implications. You have the right to not answer any question if you feel any discomfort.

This instrument is to be completed by adolescents. We assure you that all the information gathered will be anonymous and will only be used for scientific purposes.

1. Age (in years) __________
2. Gender: Male Female
3. Birthplace: Qatar Abroad (specify)_
4. Nationality: Qatari Non-Qatari (specify)___________
5. School name ______________________________
6. School type:

- Public
- Private
- others (specify)____________

7.

- How many hours do you use digital technology for **study** purposes **daily** on **weekdays (Sunday – Thursday)**?
- How many hours do you use digital technology for **non-essential reasons** **daily** on **weekdays** **(Sunday – Thursday)**?
- How many hours do you use digital technology for **study** purposes **daily** on **weekends (Friday and Saturday)?**
- How many hours do you use digital technology for **non-essential reasons** **daily** on **weekends (Friday and Saturday)?**

8. Answer the following question about your use of technology for **non-essential reasons**, please:

|  | Yes | No |
| --- | --- | --- |
| Do you feel preoccupied with the Internet (think about previous online activity or anticipate next online session)? |  |  |
| Do you feel the need to use the Internet with increasing amounts of time in order to achieve satisfaction? |  |  |
| Have you repeatedly made unsuccessful efforts to control, cut back, or stop Internet use? |  |  |
| Do you feel restless, moody, depressed, or irritable when attempting to cut down or stop Internet use? |  |  |
| Do you stay online longer than originally intended? |  |  |
| Have you jeopardized or risked the loss of significant relationship, task, educational opportunity because of the Internet? |  |  |
| Have you lied to family members, teachers, or others to conceal the extent of involvement with the Internet? |  |  |
| Do you use the Internet as a way of escaping from problems or of relieving a negative mood (e.g., when feelings helplessness, guilt, anxiety, depression)? |  |  |

9. Now we would like to know more about any problems you have had with feeling tired, weak or lacking in energy in the last month. Please answer ALL the questions by ticking the answer which applies to you most closely. If you have been feeling tired for a long while, then compare yourself to how you felt when you were last well. Please tick only one box per line. Four options for each statement ‘better than usual=1’, ‘no more than usual=2’. ‘worse than usual’=3, ‘much worse than usual=4’.

|  | **1** | **2** | **3** | **4** |
| --- | --- | --- | --- | --- |
| Do you have problem with tiredness? |  |  |  |  |
| Do you need to rest more? |  |  |  |  |
| Do you feel sleepy or drowsy? |  |  |  |  |
| Do you have problems starting things? |  |  |  |  |
| Do you start things without difficulty but get weak as you go on? |  |  |  |  |
| Are you lacking in energy? |  |  |  |  |
| Do you have less strength in your muscle? |  |  |  |  |
| Do you feel weak? |  |  |  |  |
| Do you have difficulty concentrating? |  |  |  |  |
| Do you have problems thinking clearly? |  |  |  |  |
| Do you make slips of the tongue when speaking? |  |  |  |  |
| Do you find it more difficult to find the correct word? |  |  |  |  |
| How is your memory? |  |  |  |  |
| Have you lost interest in the things you used to do? |  |  |  |  |

1. **Survey – Arabic version**

**الاستخدام المفرط للتكنولوجيا الرقمية – مشروع بحثي**

**استبيان اليافعين**

عزيزي المشارك

ندعوكم للمشاركة في دراسة بحثیة بعنوان "**الاستخدام المفرط للتكنولوجيا الرقمية وتأثيره على الصحة والتعليم والترابط الأسري في قطر"**. أنت تشارك في هذه الدراسة على أساس تطوعي. يمكنك الانسحاب من الدراسة في أي وقت دون الحاجة إلى إبداء الأسباب ودون أي تبعات. لديك الحق في عدم الإجابة على أي سؤال إذا شعرت بعدم ارتياح. هذا الاستبيان مخصص لليافعين من سن 10- 16. ونؤكد لك أننا لن نجمع أي معلومات تدل على إسمك أو شخصيتك كما نؤكد لك أن هذا الاستبيان سيستخدم لأغراض علمية فقط.

1. العمر (بالسنوات): -------
2. الجنس: ذكر أنثى
3. مكان الميلاد: قطر خارج قطر (يرجى ذكر الدولة) ---------
4. الجنسية: قطري غير قطري (يرجى ذكر الجنسية) --------
5. اسم المدرسة:
6. نوع المدرسة:

- حكومي
- خاص
- أخرى (حدد) ----------
- كم ساعة تستخدم التكنولوجيا الرقمية **لأغراض الدراسة** **يوميًا** في **أيام الأسبوع** **(الأحد - الخميس)**؟
- كم ساعة تستخدم التكنولوجيا الرقمية **لأسباب غير ضرورية** **يوميًا** في **أيام الأسبوع (الأحد - الخميس)**؟
- كم ساعة تستخدم التكنولوجيا الرقمية **لأغراض الدراسة** **يوميًا** في **عطلات نهاية الأسبوع (الجمعة والسبت)**؟
- كم ساعة تستخدم التكنولوجيا الرقمية **لأسباب غير ضرورية** **يوميًا** في **عطلات نهاية الأسبوع (الجمعة والسبت)**؟

1. أجب عن السؤال التالي حول استخدامك للتكنولوجيا **لأسباب غير ضرورية**، من فضلك:

|  | نعم | لا |
| --- | --- | --- |
| هل تشعر بأنك مشغول الذهن تجاه ما يجري في الإنترنت (أن تفكّر في نشاط سابق عبر الإنترنت أو تترقب استخدامك المقبل له )؟ |  |  |
| هل تشعر بالحاجة إلى زيادة استخدامك للإنترنت لفترات زمنية أطول من أجل تحقيق الرضا؟ |  |  |
| هل بذلت جهودًا م بشكل متكرر للتحكم في استخدام الإنترنت أو تقليصه أو إيقافه لكنها فشلت ؟ |  |  |
| هل تشعر بالقلق، أو بتقلب المزاج، أو الاكتئاب، أو الانفعال عند محاولتك تقليل أو إيقاف استخدام الإنترنت؟ |  |  |
| هل تبقى متصلاً بالإنترنت لفترة أطول مما كنت تنوي في الأصل؟ |  |  |
| هل عرضت للخطر أو خاطرت بفقدان علاقة مهمة أو فرصة تعليمية بسبب الإنترنت؟ |  |  |
| هل أخفيت الحقيقة على أفراد الأسرة أو المعلمين أو غيرهم لتجنب قول مدى استخدامك للإنترنت؟ |  |  |
| هل تستخدم الإنترنت كطريقة للهروب من المشاكل أو للتخلص من مزاج سلبي (على سبيل المثال، عند الشعور بالعجز، والشعور بالذنب، والقلق، والاكتئاب)؟ |  |  |

1. نود الآن معرفة المزيد عن أي مشاكل واجهتها مع الشعور بالتعب أو الضعف أو نقص الطاقة في الشهر الماضي. الرجاء الإجابة على **جميع** الأسئلة عن طريق تحديد الإجابة التي تنطبق عليك بأكبر قدر. إذا كنت تشعر بالتعب لفترة طويلة، فقارن نفسك بأخر مرة شعرت فيها أنك على ما يرام. الرجاء تحديد مربع واحد فقط في كل سطر. هناك أربعة خيارات لكل عبارة: "أقل من المعتاد = 1"، "ليس أكثر من المعتاد = 2"، "أسوأ من المعتاد" = 3، "أسوأ بكثير من المعتاد = 4".

|  | 1 | 2 | 3 | 4 |
| --- | --- | --- | --- | --- |
| هل لديك مشكلة مع التعب؟ |  |  |  |  |
| هل تحتاج إلى المزيد من الراحة؟ |  |  |  |  |
| هل تشعر بالنعاس أو الدوار؟ |  |  |  |  |
| هل لديك مشاكل في بدء عمل الأشياء؟ |  |  |  |  |
| هل تبدأ عمل الأشياء دون صعوبة ولكن تضعف كلما تقدمت في العمل ؟ |  |  |  |  |
| هل تشعر أنك تفتقر إلى الطاقة؟ |  |  |  |  |
| هل لديك قوة أقل في عضلاتك؟ |  |  |  |  |
| هل تشعر بالضعف؟ |  |  |  |  |
| هل تجد صعوبة في التركيز؟ |  |  |  |  |
| هل لديك مشاكل في التفكير بوضوح؟ |  |  |  |  |
| هل ترتكب زلات لسان عند التحدث (أي قول كلمة أو عبارة تندم عليها)؟ |  |  |  |  |
| هل تجد صعوبة أكثر في العثور على الكلمة الصحيحة |  |  |  |  |
| كيف هي قابليتك على التذكر؟ |  |  |  |  |
| هل فقدت الأهتمام بالأشياء التي اعتدت القيام عليها؟ |  |  |  |  |

**3. Scatter plots for fatigue types with predictors**

Figure S1 provides additional information on the associations between fatigue scores and the independent variables of age, essential use, non-essential use and total IA.


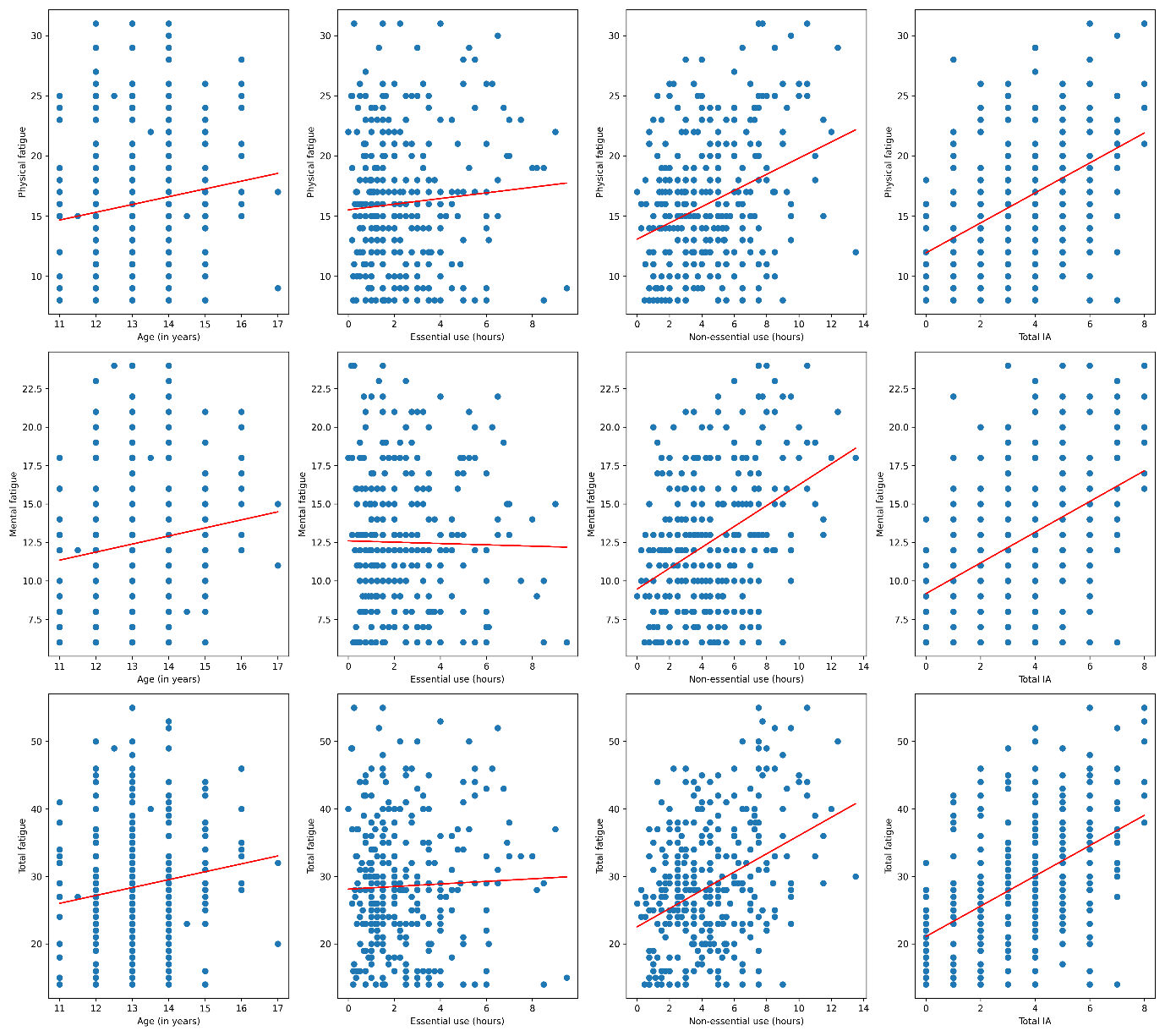


Figure S1. Scatter plot grid for the three fatigue scores measured against the independent variables (predictors)
